# Shape-Up and Eat Right Families Pilot Program: Feasibility of a Weight Management Shared Medical Appointment Model in African-Americans With Obesity at an Urban Academic Medical Center

**DOI:** 10.3389/fped.2018.00101

**Published:** 2018-04-12

**Authors:** Gitanjali Srivastava, Kenya D. Palmer, Kathy A. Ireland, Ashley C. McCarthy, Kate E. Donovan, Aaron J. Manders, Juhee McDougal, Carine M. Lenders, Caroline M. Apovian

**Affiliations:** ^1^Nutrition and Weight Management Research Center, Boston Medical Center, Department of Medicine, Section of Endocrinology, Diabetes, Nutrition and Weight Management, Boston University School of Medicine, Boston, MA, United States; ^2^Nutrition and Fitness for Life Program, Boston Medical Center, Department of Pediatrics, Boston University School of Medicine, Boston, MA, United States; ^3^Department of Medicine, Section of General Internal Medicine, Boston University School of Medicine, Boston, MA, United States

**Keywords:** shape-up and eat right families, weight loss in ethnic minorities, shared medical appointments, family-based weight loss, disparities to obesity care, maternal-child health, childhood obesity

## Abstract

**Objectives:**

Disparities in obesity care exist among African-American children and adults. We sought to test the feasibility of a pilot program, a 1-year family-based intervention for African-American families with obesity [shape up and eat right (SUPER)], adopting the shared medical appointment model (SMA) at an urban safety net hospital.

**Outcomes:**

Primary outcomes: (1) family attendance rate and (2) program satisfaction. Secondary outcomes: change in body mass index (BMI), eating behaviors, and sedentary activity.

**Methods:**

Adult parents (BMI ≥ 25 kg/m^2^) ≥18 years and their child(ren) (BMI ≥ 85th percentile) ages 6–12 years from adult or pediatric weight management clinics were recruited. One group visit per month (*n* = 12) consisting of a nutrition and exercise component was led by a nurse practitioner and registered dietitian. Height and weight were recorded during each visit. Participants were queried on program satisfaction, food logs and exercise journals, Food Stamp Program’s Food Behavior, and the Expanded Food and Nutrition Education Program food checklists.

**Results:**

Thirteen participants from lower socioeconomic zip codes consented [*n* = 5 mothers mean age 33 years, BMI of 47.4 kg/m^2^ (31.4–73.6 kg/m^2^); *n* = 8 children; mean age 9 years, BMI of 97.6th percentile (94–99th percentile); 60% enrolled in state Medicaid]. Average individual attendance was 23.4% (14–43%; *n* = 13); monthly session attendance rates declined from 100 to 40% by program completion; two families completed the program in entirety. Program was rated (*n* = 5 adults) very satisfactory (40%) and extremely satisfactory (60%). Pre-intervention, families rated their eating habits as fair and reported consuming sugar-sweetened beverages or sports drinks, more so than watching more than 1 h of television (*p* < 0.002) or video game/computer activity (*p* < 0.006) and consuming carbonated sodas (*p* < 0.004). Post-intervention, reducing salt intake was the only statistically significant variable (*p* < 0.029), while children watched fewer hours of television and spent less time playing video games (from average 2 to 3 h daily; *p* < 0.03).

**Conclusion:**

Attendance was lower than expected though children seemed to decrease screen time and the program was rated satisfactory. Reported socioeconomic barriers precluded families from attending most sessions. Future reiterations of the intervention could be enhanced with community engagement strategies to increase participant retention.

## Introduction

Obesity causes significant cardiovascular disease, diabetes, hypertension, and overall morbidity and mortality in the United States (US) (1, 2). Over one-third of US adults are afflicted with obesity (35% men and 40% women) and specifically women, not men, had worsening disease severity and progression to class III [body mass index (BMI) ≥40 kg/m^2^] between 2005 and 2014 (3). About 18 and 7.9% of children and adolescents have obesity and severe obesity (≥120% of 95th percentile for BMI; class II and class III obesity) respectively (4). There also has been a rise in obesity in adolescents between the ages of 16–19 years with 41.5% having obesity and 4.5% meeting criteria for class III obesity (>140% of 95th percentile for BMI); children ages 6–11 years have had similar increase in obesity prevalence to 37.3% with respective 12.8% meeting criteria for class II and III obesity (4). In particular, in the US, the epidemic is marked by racial and ethnic disparities: non-Hispanic Black (15.8%) adolescents have the highest prevalence of this disease than non-Hispanic Whites (13.1%), non-Hispanic other race or multi-race (10.9%), and Hispanic (15.2%) adolescents with obesity (5). A lower percentage of non-Hispanic Blacks (35%) seek weight loss treatments compared to their non-Hispanic White counterparts (39%) (6); non-Hispanic Blacks are also more likely to avoid care (OR 0.49, 95% CI 0.26–0.90) and less likely to initiate weight loss discussion with their providers than non-Hispanic Whites (7). Persistent barriers and disparities to care among racial/ethnic minorities exist likely due to a combinatory effect of social, economic, biological, and environmental factors affecting macro- and micro-environments (8) with US medical professionals not adept or trained at addressing the complexity of care and higher attrition rates for medical weight loss visits (9–12).

As a result, it becomes more essential to evaluate novel effective obesity-care delivery models among various populations. In regards to chronic diseases and obesity, shared medical appointments (SMAs) are innovative, patient-receptive, cost-effective methods of clinical practice (13–16) with a family-based approach particularily utilized for pediatric interventions, such as in childhood obesity (17, 18). SMAs are a redesign of chronic care models where groups of patients (8–20) are seen by a multidisciplinary team in 1 h in efforts to improve clinic throughput and efficiency and have shown improved cardiovascular benefit and quality improvement in diabetes management (19). These group-based patient visits address the gaps in knowledge, skills, and support necessary to maintain overall health and well-being, while increasing the time allocated to discuss lifestyle modification and healthy behaviors, especially in the context of a busy clinical practice where an individual visits may last only 15–20 min (13). Moreover, in an effort to increase accessibility to care and address disparities, SMAs have been adopted as novel methods to address those needs among healthcare systems (20). However, to date, SMAs have not been studied in challenging or culturally relevant populations, such as in African-Americans, where attrition rates are typically higher and patients often present with decreased literacy, non-adherence to recommendations, and transportation concerns, along with patient–physician weight bias, all of which present remarkable barriers to effective treatment for obesity (21–23). SMAs may provide burgeoning solutions to more effective care for these populations in the long-term through group engagement, where individuals may identify themselves with others of similar background and through use of family friendly tools to enhance motivational change. Furthermore, previous studies evaluating group medical visits have shown the shift in focus of the provider to that of an adjudicator advocating self-care norms based on medical knowledge in the context of the patient’s lived experience and subsequently the patient disseminating these norms to others within the family unit (24). Medical group visits may have tangible benefits to bridging challenging social needs, complex chronic diseases, and improving patient–provider experiences (25). Additionally, SMAs are typically age-specific, targeting either adult or pediatric patients (but not both) with specific disease process or health goals, rather than being an all-inclusive design with common health targets. Thus, we sought to determine feasibility of a unique monthly parent–child-based weight loss intervention among African-American families utilizing a novel family centered SMA model at an adult and pediatric weight management center associated with an urban safety net academic hospital.

## Materials and Methods

Shape-up and eat right (SUPER) families was a pilot program to identify the feasibility of a family-based multidisciplinary group program for weight loss at Boston Medical Center (BMC) over a period of 1 year to foster healthy eating and exercise patterns for the entire family. The study was a collaboration between two weight management programs at BMC: a pediatric program, the nutrition and fitness for life (NFL) and an adult program, the nutrition and weight management center (NWMC). The primary outcomes were: (1) family attendance rate and (2) satisfaction with the program. Secondary outcomes included (1) change in BMI and (2) change in eating and sedentary behaviors.

### Recruitment

The study was approved by the BMC/Boston University Medical Campus institution review board. It was supported by the Vela Foundation. Inclusion criteria were as follows: African-American parents ≥18 years from the NWMC with either overweight or obesity (BMI ≥ 25 kg/m^2^) with one or more children aged 6–12 years with at least overweight status (BMI ≥ 85th percentile for weight and height based on the Centers for Disease Control (CDC)′s BMI Percentile Calculator for Child and Teen [http://nccd.cdc.gov/dnpabmi/]) or if the parent and/or guardian from the NWMC had a child currently being treated at the NFL program or if a child from NFL had a parent with overweight or obesity who may have not been part of NWMC. Exclusion criteria were as follows: (1) families not currently enrolled in either NFL or NWMC, (2) primary language other than English, (3) inability or not willing to provide informed consent/assent, (4) ethnicity other than African-American, and (5) non-overweight or obesity in adult or child. Informed consent was obtained from parents of children at the time of enrollment and children aged 6–12 years provided assent. Once consent was obtained, all contact information was updated. The initial contact was made in the respective clinic or telephone based. Demographic information querying zip code, race, ethnicity, level of education, primary source of income, employment status, insurance coverage, health of household members, and number residing in the household were collected. An abridged self-reported physical activity questionnaire modified from PAQ-C and PAQ-A (26) was also completed by telephone, assisted by the nurse practitioner, with the purpose of guiding exercise education and physical activity component of the group sessions. The participants were then notified of the first group visit by phone, email, and/or postal mail. The pilot program aimed to enroll at least six families (at least one adult parent/guardian and one child) based on allocated funding from the Vela Foundation. Because families were recruited directly from either NFL or NWMC and this was a feasibility study, there was no randomization. To protect the children participating in this study, children were not separated from the parents. All materials provided to the children were “child-friendly,” while adult materials were written at a fifth grade reading level. All program visits occurred at BMC.

### Monthly Group Visits (Visit #1–11)

There were 12 monthly 60–90 min sessions scheduled over a period of 1 year. A nurse practitioner with a Masters in Exercise Science, registered dietitian, and research coordinator were present at each group. A routine medical intake was completed by the nurse practitioner during the initial visit. Attendance was recorded at each visit. During monthly group visits, anthropometric measurements (height/weight) were taken for both adults and children. The Food Stamp Program’s Food Behavior Checklist (FBC) (27), which measures basic dietary behaviors, including frequency of fruit, vegetables, juice and low-fat dairy consumption, food insecurity, shopping, and cooking techniques, was administered at the first and last group session to assess changes in dietary behavior. A second questionnaire, The Expanded Food and Nutrition Education Program (EFNEP) Food Checklist (28–31), which evaluates the way families plan and prepare food and assesses sedentary and screen-time behaviors in children was also administered during the first and last group session. Adult participants were encouraged to use a free pedometer application if they had a smartphone; all families were provided a pedometer. All participants were given a food journal and a log to record their daily steps as well as a physical activity goal-setting worksheet. Degree of satisfaction was assessed using a validated five-point satisfactory Likert scale (32) at the end of each group visit and the end of the program. A mid-point survey was collected at 6 months to determine if program adjustments were needed. Families were contacted *via* telephone, email, and postal mail between visits.

Group visits included topics for nutrition, exercise, and stress management. Sessions began with a 20–30 min of exercise training (body weight/calisthenics) including moderate cardiovascular exercise led by a nurse practitioner trained in exercise science. Physical activity and exercise education were adapted from the guidelines promoted by the American College of Sports Medicine (ACSM) and the CDC. Workouts included a dynamic warm up and a combination of calisthenics (burpees, alligator walks, kicks, jumps) and body weight exercises (push-ups, lunges, and plank exercises). Participants were monitored by clinical study staff for any exercise-related adverse events and were asked to report during and after exercise if they were experiencing any chest pain, dizziness, leg pain, calf pain, or hip pain. Families were expected to do these exercises on their own as a family for 2–3 times per week with written explanations of the workouts provided upon request. Families were also expected to perform moderate-to-vigorous cardiovascular exercise 2–3 times weekly to achieve the ACSM/CDC recommended minimal levels of physical activity weekly. Specific, measurable, attainable, relevant/realistic, time-based short-term and long-term goal worksheets were also provided to patients during each visit, along with pictures and description of potential exercises which could be conducted within the home without the need for access to a gym, to help improve dietary intake and physical activity.

The second part of each session was devoted to nutrition education and counseling led by the dietitian. This included the basics of healthy eating (macronutrient content and calories), options for navigating dietary changes with children, plus optional healthy cooking demonstrations biweekly located at the demonstration kitchen on campus. The nutrition curriculum utilized in this pilot study was adapted from myplate.gov “10 Tips Nutrition Education series” and developed by NFL’s registered dietitians. Information was adapted and provided in an age appropriate format.

Attendance was calculated by totaling the number of group visits completed, divided by the number of group visits offered. Satisfaction was measured *via* survey using a 1–5 Likert scale (32). Weight status was measured by comparing the height and weight from baseline to the end of the program. SPSS program was used for descriptive statistical analysis of the data.

### Final Visit (Visit #12)

The final SUPER-families visit concluded the study. During this visit, families completed a final EFNAP and FBC program satisfaction questionnaires; and final heights and weights were recorded. Participants were also asked to identify long-term family dietary, nutrition, and/or stress-related goals. FitBits^®^ were distributed as a reward for completion of the program.

### Retention

Families were sent at least three reminder phone calls from study staff and letters prior to each group visit. If a family missed a session, every effort was made to contact the family, identify the reason, and encourage participation in the next session.

## Results

### Demographics

Though a total of initial six families consented to participation, one family canceled on start date. Thus, a total of five families [five adult parents (all mothers though fathers and grandparents/and or guardians were also invited)] and eight children consented to the program and were present at initiation of the intervention. Adult average age was 33 years with average BMI of 47.4 kg/m^2^ (31.4–73.6 kg/m^2^). Average age for the children was 9 years with average BMI of 97.6th percentile (94th–99th percentile). All participants were of African-American descent living in an urban metropolitan area in lower socioeconomic zip codes; 60% of participants were enrolled in Mass Health Medicaid program. The highest educational degree attained by any of the parents was a college degree (*n* = 2); one family’s source of child support was food stamps. All of the families spoke English as their primary language.

### Primary Outcomes: Attendance Rates and Program Satisfaction

Attendance was lower than expected. Three families completed some of the sessions and two families completed the entire 12-month program. Individual attendance ranged from 14 to 43% (average 23.4%; *n* = 13) with session attendance rates declining from 100 to 40% by the end of the program. Parents were queried about barriers to attendance; the perceived barriers reported by 60% of the families included time conflicts due to other family member activities, transportation, loss of employment/housing, moving, new birth, and lack of personal time. Satisfaction survey (*n* = 5 adults) showed that 40% of the parents were very satisfied and 60% were extremely satisfied with the monthly sessions.

### Secondary Outcomes: Change in BMI, Eating, and Sedentary Behaviors

**Figure 1:**
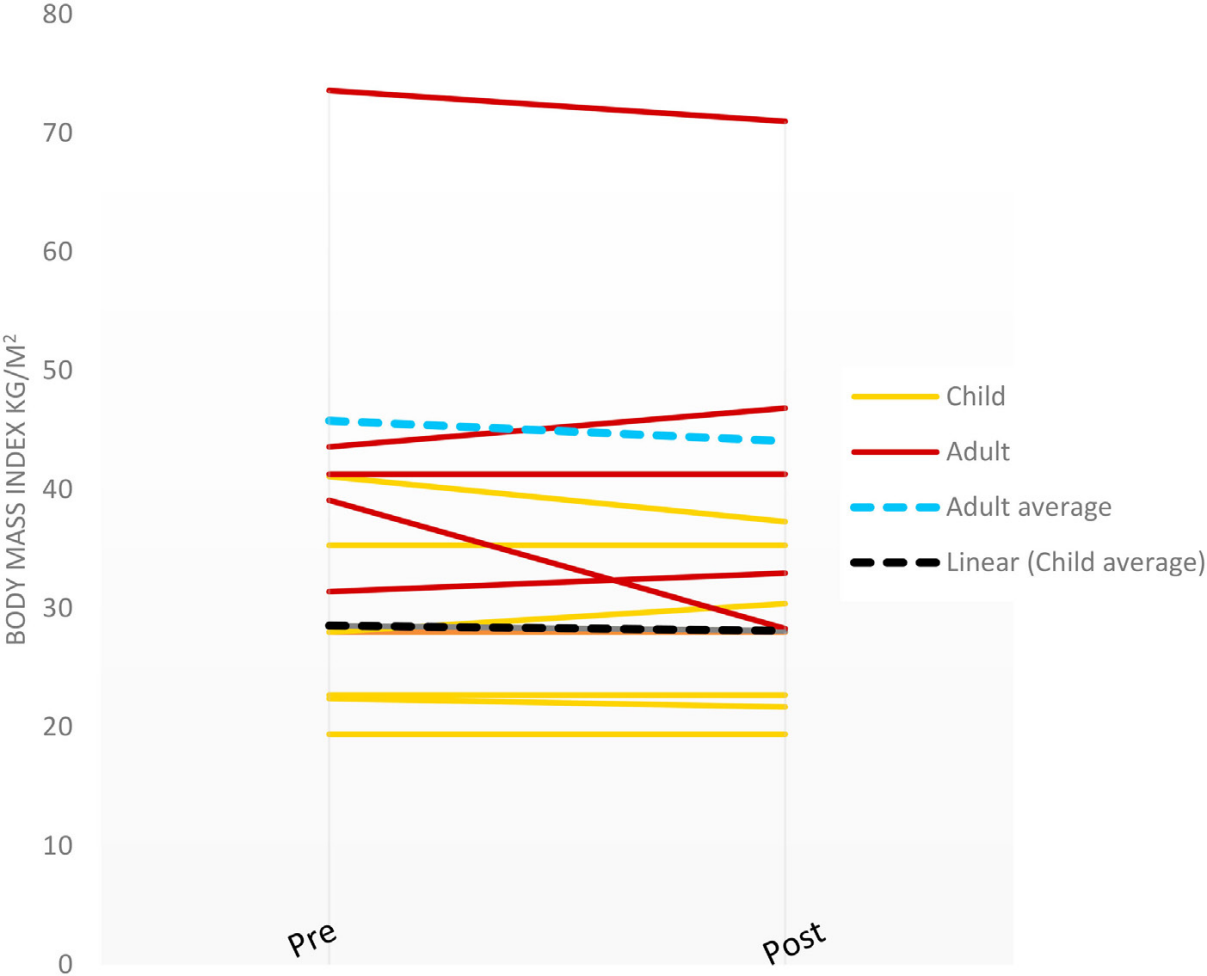
Shape-up and eat right families body mass index changes pre- and post-intervention (last observation carried forward).

Change in BMI (Figure 1) was calculated for the families completing the study. Adult mothers had an average BMI decrease of −1.71 kg/m^2^ (range: −10.0 to 3.26 kg/m^2^) and the children experienced an average BMI decrease of −2.6 kg/m^2^ (range: −3.8 to −1.4 kg/m^2^). There were no statistically significant differences in these outcomes, however (*p* > 0.05). Changes in eating behaviors were assessed based on families completing both pre- and post-questionnaires, food journals, and written feedback at the end of the sessions.

### Eating and Food Behaviors Questionnaires

**Figure 2:**
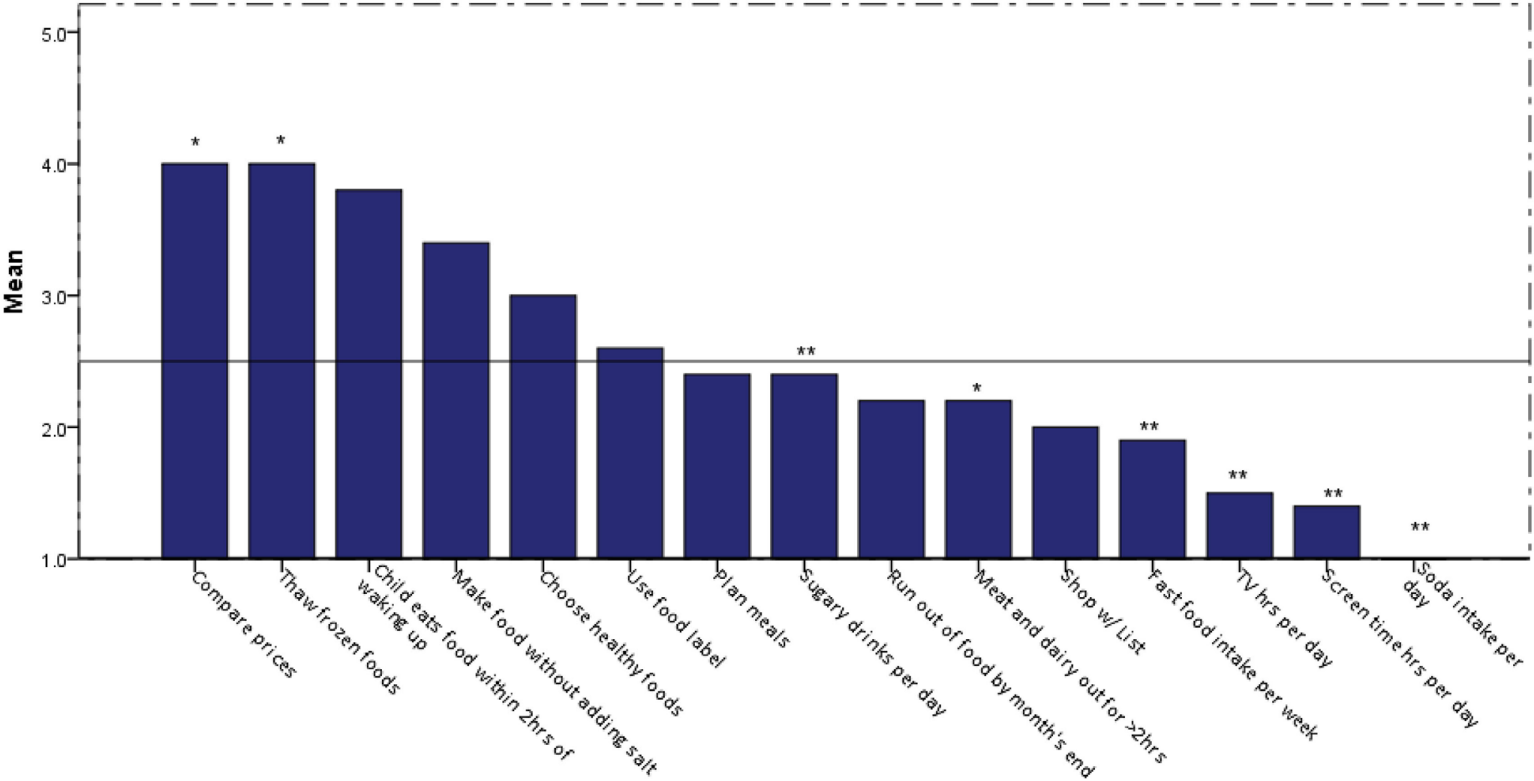
Eating behaviors of shape-up and eat right-families prior to intervention. Legend: 1 = no, 2 = sometimes, 3 = often, 4 = very often, 5 = almost always; **Frequency on *Y*-axis corresponds to number variable; *p < 0.05.

**Figure 3:**
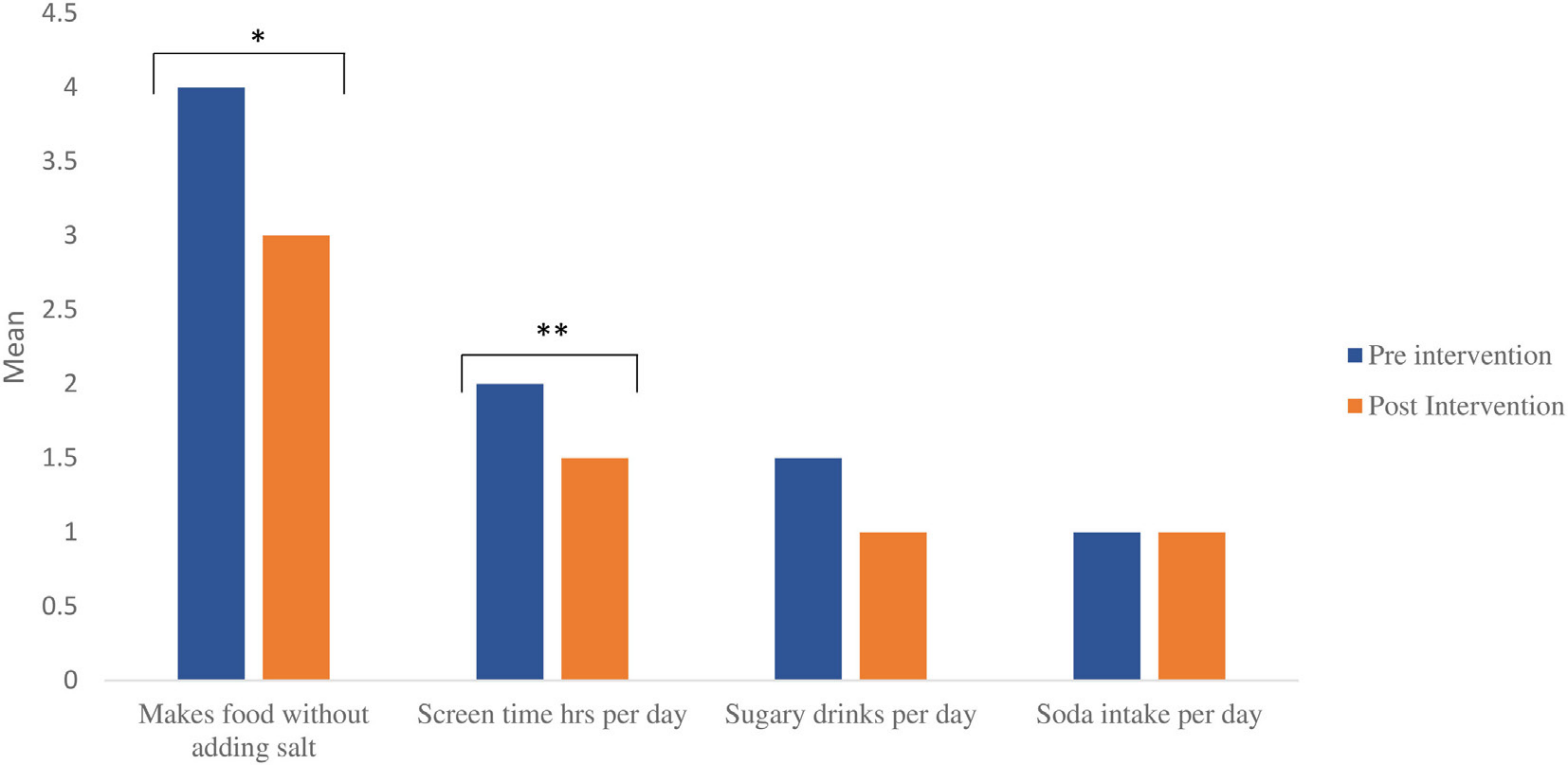
Food and nutrition behaviors of shape-up and eat right families pre- and post-intervention (data for program completers). Legend: 1 = no, 2 = sometimes, 3 = often, 4 = very often, 5 = almost always; *p = 0.029, **p = 0.034; for sugary drinks and soda intake, value on *Y*-axis corresponds with numeric value per day.

Prior to the intervention, families reported drinking milk often, eating an average of at least one serving of fruit and vegetable per day, having an average of at least one serving of fish daily, and rating their eating habits as fair (average rating 3.1, 1 = poor; 10 = excellent); the differences in these reported behaviors post-intervention were not statistically significant. Upon initial visit, families were less likely to have meat or dairy sit out for more than 2 h (*p* < 0.002) or thaw frozen foods at room temperature (*p* < 0.0005; Figure 2). Comparing prices was the highest variable affecting eating behaviors among the families with an inclination toward choosing healthy foods for the family (Figure 2). Planning meals, shopping with a list, and reading food labels were less likely to occur among the families. Most of the families had children who ate within 2 h of waking up. Families reported consuming 2.4 servings daily of sugar-sweetened beverages or sports drinks, more so than watching more than 1 h of television (*p* < 0.002) or video game/computer activity (*p* < 0.006) and consuming carbonated sodas (*p* < 0.004). The consumption of carbonated sodas (EFNEP checklist provided images of carbonated sodas on the questionnaire) was low among all of the families [an answer of “1” (serving) was reported]. No statistically significant changes were noted among frequency of sugar-sweetened beverage or soda consumption (Figure 3).

Reducing table salt when preparing food post-intervention was statistically significant (*p* < 0.029; data for families pre- and post-survey completers; Figure 3). Results from the EFNEP Food Checklist also showed that children watched fewer hours of television and spent less time playing video games (from average 3 to 2 h daily; *p* < 0.03 for survey completers; Figure 3) and reported an increase in sports participation after the intervention. No statistically significant changes were noted among frequency of sugar-sweetened beverage or soda consumption based on questionnaire results (Figure 3).

### Food Journals and Written Feedback

Though the questionnaires post-intervention did not show statistically significant changes associated with frequency of sugar-sweetened or carbonated beverage consumption, patient-reported changes in behavior based on food journals, and written feedback were favorable indicating a change to ingesting less fruit drinks, sports drinks, or punch and increasing servings of fruit from 1 to 2 per day. Families reported healthier food habits, including adding more fish to their diet, reading labels more often, and increased meal planning. Families planned to exercise more frequently together. Families also reported being very satisfied with the sessions and were very hopeful the program would continue in the future.

### Physical Activity

Though pedometers were distributed to each family member with instructions to self-document step count on goal worksheets provided, participants did not record their step count and forgot to bring their pedometers during the visits. Thus, physical activity data was not quantifiable.

## Discussion

A parent–child group intervention for the treatment of overweight and obesity among African-American families had low attendance despite being deemed satisfactory by enrollees. The intervention was designed specifically taking disparities to care into account such as level of education (materials designed at fifth grade reading level), occurrence during evening hours to accommodate work/school schedules, and all-inclusive free visits, including accessibility to dietitians and medical providers on-site. A program fee was also not charged to any of the participants. Those who attended were likely quite motivated and thus rated the program as satisfactory. Parents were queried about barriers to attendance and reasons for this low affluence included several socioeconomic concerns; primary stability and sustainability of the family unit barred from attending medical group visits in the SUPER-families. The lack of participation and overall completion rate of this program reinforces current research noting and addressing significant barriers to care and health disparities among African-Americans (33–36).

In our study, the SMA model was within a traditional clinical academic urban hospital setting, rather than outsourced or integrated within a community or neighborhood. Barriers predominately at the socioeconomic and community level precluded attendance at the sessions and suggest that group-based interventions such as SUPER-families will likely be more successful in a community-based neighborhood setting with closer proximity and improved accessibility to care adopting the SMA model. However, probably more important than placement of these SMAs in community-based locations, is community engagement to determine strategies that might work in enhancing attendance. Though barriers to attendance were queried, parents were not queried on strategies to help overcome low attrition and attendance rates, which should be included in future reiterations of this intervention. Furthermore, though the urban inner-city academic hospital where the study took place serves a diverse racial/ethnic minority underserved population, efforts to engage the community in recruitment of community-based participatory research needs attention. A previous study from this institution has cited challenges in recruitment for pediatric obesity trials even with financial incentives (out of 179 eligible, only 4 attended the visits) (37). Recently, the Communication, Awareness, Relationships, and Empowerment (C.A.R.E.) model to engage at-risk African-American population in the Washington, DC area cultivated not only a visible presence at community events, but also through partnerships with churches, health organization, academic institutions, and governmental agencies, was able to successfully recruit and retain the study participants (38). Other strategies have included establishment of a community advisory board for at-risk populations in the metro area, which was able to conduct focus groups and pilot web-based and wearable technology through members representing churches in a cardiovascular health and needs assessment study (39). Community-based interventions have been shown to promote weight loss or positively influence diet and physical activity behavior in a culturally congruent manner, such as in churches or school-based programs, among African-Americans (40–43). For example, recently churches have been recognized as powerful outreaches for health services and interventions in an effort to address disparities among lower income, African-Americans families given the pastors’ view on holistic health with integrated faith, such as their perspectives on obesity and infant mortality (44). Scalable, community-based weight loss programs with combination interventions, such as portable kiosks for assessments, email/text supports, and online-health coaches utilized in the Weigh and Win study of 40,038 adults, which combine outreach, effectiveness, and costs may help reduce disparities among African-Americans (45). Other studies have shown some benefit with incorporation of other resources such as tailored, interactive text-messages to enhance weight loss success among African-Americans with obesity (46). The use of a community-based participatory research approach to recruit African-Americans into pilot interventions and innovative health promotion programs has demonstrated success and feasibility along with scalability of the intervention as it applies to minority at-risk populations (47). Thus, though this current pilot SUPER-families SMA model was not successful in a hospital-based setting due to attrition and retention, programmatic elements could be better applied using the C.A.R.E. model for community engagement.

Though not statistically significant, it is noteworthy that the pilot study suggested an improvement of an average BMI change of −2.6 kg/m^2^ in the children post-intervention. The study possibly suggests that this type of parent–child group-based intervention may potentially have long-term benefits for younger children with obesity. The education provided during the intervention did improve healthy living behaviors for all participants involved without determining overall effects on weight. Children may perhaps be more receptive to an intensive group-based education and weight loss intervention when paired with a parent/and or guardian. Moreover, the intervention improved sedentary time and decreased screen time behaviors for the children involved in the study. Of important consideration is that the rate of severe obesity has significantly increased in pre-school age children 2–5 years, since the 2013–2014 cycles (4). Because childhood obesity correlates closely with adolescent and adult obesity leading to worsening disease severity when older (48–51), and because children have a natural affinity toward their mothers, this SMA pilot intervention utilizing the mother–child relationship has a potential prospective of being adopted in pregnancy with a focus on prevention, rather than treatment. Early nutrition and growth in the initial years of life are important determinants of later body weight and metabolic health (52–54). Therefore, future SUPER-family models may consider preventive strategies targeting either pregnant African-American women or younger children ages 2–5 years, rather than 6–12 years old.

Furthermore, the pilot study, though a small number of enrolled participants, suggests an improvement in overall basic education around nutrition, eating, and sedentary behaviors with increased likelihood of implementation of these positively learned behaviors in the future, especially with regards to reducing salt intake and decreasing screen (video/computer) time. Reported screen time >3 h independent of physical activity has been associated with cardiometabolic risk factors and high BMIs in children (55, 56). Also noteworthy that these two behaviors (reduced salt intake and screen time) were simple, inexpensive, and could be implemented without difficulty and without much disruption to regular household duties, reinforcing that obesity interventions most likely need to be simplified in this specific population. Additionally, cost of food was of highest concern for the SUPER mothers though they preferred healthier foods. Recent studies have highlighted higher costs of healthier foods (57, 58), which could potentially be a challenging factor adversely affecting nutrition among African-American families. Research around understanding alternative healthier, affordable food patterns in this population to alleviate disparities to nutrition is recommended. Interestingly, differences in consumption of sugary beverages pre- and post-intervention in the study were not statistically significant though participants reported an average consumption of 2.4 drinks per day. A decrease in soda consumption could have led to cost savings, and the possibility of participant underreporting pre-intervention should be considered. Recent efforts discouraging sodas consumption from a public policy standpoint and overall public awareness (59–61) may have resulted in participant stigmata when answering the question relevant to beverage consumption. Because high fructose corn syrup found in sugary beverages has been well known to correlate with higher childhood adiposity and detrimental effects on metabolism (62, 63), more emphasis on avoidance of sugary beverages may be needed in future SUPER-families studies including clarifying the families’ perception and understanding of these beverages given the negative statistical significance on the questionnaires compared to the food journal and written feedbacks.

Last, the study has several limitations. First, because the primary objective of the study was to estimate rate of participation, drop-out, or compliance, with a pre-determined number of minority families (*n* = 6) recruited based on funding allocated, data has limited interpretation due to small sample size, high attrition, and low retention rates, and cannot be generalizable to a whole population. A robust sample size is likely needed to determine overall effects of the study on primary and secondary outcomes. In addition, self-reported data on food journals, logs, and written feedback likely contain potential bias due to exaggeration and attribution. The families enrolled were already seeking treatment for weight loss prior to enrollment into the study and preselected without randomization. Thus any failure to lose weight may have been attributed to their perceived barriers to success and positive nutrition or physical activity behaviors exaggerated. Also, the study emphasized maternal–child pair, rather than father–child, or coparent–child relationship though fathers, grandparents, and/or legal guardians of the children were also invited to be part of the study. The latter relationships may yield different results. All adults in the study were mothers through self-selection and motherhood may reflect a greater role in child healthcare among this population. Though the study was done in African-Americans, the study cannot be generalized to other racial and/or ethnic minorities. Future improvements could also tailor culturally relevant exercises, rather than body weight and calisthenics conducted in the study.

## Conclusion

In conclusion, although the study demonstrated an overall benefit in children more than adults in this particular population and was deemed satisfactory by enrollees, a family-based SMA intervention for weight loss in African-American families does not address barriers precluding high attrition rates for monthly nutrition and exercise visits, and would be more successful if applied in a field-based community setting. Despite acknowledging satisfaction and barriers half-way through the program, changes made were not enough to overcome barriers to care experienced by African-Americans in urban communities. Future research is needed to develop novel ways to deliver knowledge on nutrition and exercise, valued by families within a group program such as the one designed in this pilot study. New programs may include community-based locations, home visits, and web-based interventions to disseminate nutrition and physical activity education and tools in an effort to eliminate barriers to care and improve health outcomes as they relate to overweight and obesity within the African-American community. Moreover, because community engagement perpetuates scalability of the intervention, future re-design of the intervention might consider community-based advisory boards and partnerships for participant recruitment and retention.

## Ethics Statement

This study was carried out in accordance with the recommendations of applicable federal, state, and local laws and regulations, and the relevant policies of the Human Research Protection Program, BMC, and Boston University with written informed consent from all adult subjects. All adult subjects gave written informed consent in accordance with the Declaration of Helsinki. The protocol was approved by the BMC and Boston University Medical Campus Institutional Review Board. Written informed consent was obtained from parents of children at the time of enrollment and children ages 6–12 years provided assent.

## Author Contributions

KDP, KAI, ACM, AJM, CML, and CMA contributed equally to the planning of the study, conduct of the study, analysis of the data, and intellectual input in interpretation. KED contributed significantly in conduct of the study, analysis of the data, and intellectual input in interpretation. JM and GS contributed significantly in analysis and interpretation of the results. GS contributed significantly in intellectual content of manuscript. All authors are equally responsible for accuracy and originality of this work.

## Conflict of Interest Statement

KDP, KAI, ACM, KED, AJM, JM declare no competing interests. GS reports consultant fees from Johnson and Johnson. CML received support from New Balance Foundation and BNORC P30 DK40561. CMA reports personal fees from Nutrisystem, personal fees from Zafgen, personal fees from Sanofi-Aventis, grants and personal fees from Orexigen, personal fees from NovoNordisk, grants from Aspire Bariatrics, grants and personal fees from GI Dynamics, grants from Myos, grants and personal fees from Takeda, personal fees from Scientific Intake, grants and personal fees from Gelesis, other from Science-Smart LLC, personal fees from Merck, personal fees from Johnson and Johnson, grants from Dr. Robert C. and Veronica Atkins Foundation, grants from Coherence Lab, grants from Energesis, grants from PCORI, and grants from NIH outside the submitted work.
